# A Real-Time PCR Method for the Authentication of Common Cuttlefish (*Sepia officinalis*) in Food Products

**DOI:** 10.3390/foods9030286

**Published:** 2020-03-04

**Authors:** Amaya Velasco, Graciela Ramilo-Fernández, Carmen G. Sotelo

**Affiliations:** Instituto de Investigaciones Marinas (IIM-CSIC), Eduardo Cabello 6, 36208 Vigo (Pontevedra), Spain; graciela@iim.csic.es (G.R.-F.); carmen@iim.csic.es (C.G.S.)

**Keywords:** Sepia, common cuttlefish, *Sepia officinalis*, real-time PCR (Polymerase Chain Reaction), species identification, food authentication, COI (Cytochrome Oxidase I)

## Abstract

Cephalopods are very relevant food resources. The common cuttlefish (*Sepia officinalis*) is highly appreciated by consumers and there is a lack of rapid methods for its authentication in food products. We introduce a new minor groove binding (MGB) TaqMan real-time PCR (Polymerase Chain Reaction) method for the authentication of *S. officinalis* in food products to amplify a 122 base pairs (bp) fragment of the mitochondrial COI (Cytochrome Oxidase I) region. Reference and commercial samples of *S. officinalis* showed a threshold cycle (Ct) mean of 14.40, while the rest of the species examined did not amplify, or showed a significantly different Ct (*p* < 0.001). The calculated efficiency of the system was 101%, and the minimum DNA quantity detected was 10^−4^ ng. No cross-reactivity was detected with any other species, thus, the designed method differentiates *S. officinalis* from other species of the genus *Sepia* and other cephalopod species and works for fresh, frozen, grilled, cooked and canned samples of *Sepia* spp. The method has proved to be reliable and rapid, and it may prove to be a useful tool for the control of fraud in cuttlefish products.

## 1. Introduction

Cephalopods are a very diverse group of mollusks and include 28 families and more than 600 species, many of which are commercially important. As a sign of their relevance, captures of cephalopods in 2017 reached 3,772,565 t, with an estimated value of almost 8000 million dollars [1].

The common cuttlefish (*S. officinalis*) is highly appreciated by consumers around the world, and it is traded with different presentations particularly in Japan, the Republic of Korea, Italy and Spain. In the last decade, the world catches attributed to this species have registered numbers between 20,000 and 30,000 tons every year [2]. It is the species of cuttlefish with the highest commercial value.

European regulations regarding the labeling of fishery products [3,4] establish that these products must show the information about the species, with the commercial and/or scientific name depending on the type of product. Illicit substitution of one species for another may constitute economic fraud and/or misbranding violations. Furthermore, species substitution may cause potential food safety hazards to be overlooked by processors or end-users [5]. Species substitution is relatively frequent in seafood products [6], and particularly in products containing cephalopods, where several cases of species substitution have been reported [7,8].

Species belonging to the genus *Sepia* can look very similar to a non-trained consumer, especially when they are processed for the market (e.g., peeled, canned), making the visual differentiation almost impossible and increasing the possibilities of fraud. Thus, the reported cases of mislabeling in products containing *Sepia* spp. have usually been substitutions between species belonging to the same genus [9,10]. These cases can be attributed to economic fraud (e.g., substitutions between species with different commercial value) or unintentional substitutions, which can be due to similar geographic distribution of species (e.g., *S. officinalis/S. orbignyanya/S. elegans*) and/or similar morphological characteristics (e.g., juveniles of *S. officinalis/S. elegans*) which can lead to misidentification at any level of the value chain (fisheries, processors and consumers). In order to control these substitutions, a variety of genetic methods have been published for the identification of several cephalopod species. The majority of these are labor-intensive and time-consuming, such as forensically informative nucleotide sequencing (FINS), barcoding [10,11,12,13,14] and RFLP [8,15]. Some rapid DNA-based methods have also been published for the authentication of some cephalopod species [7,16,17,18], but to date, there is not any rapid technique available for the genetic identification of *S. officinalis*.

This work presents a rapid and reliable method for the authentication of *S. officinalis* in different food matrices, including processed products. Therefore, it can be a useful tool for control authorities at different levels of the value chain.

## 2. Materials and Methods 

### 2.1. Sampling and DNA Extraction

In this work, 14 samples of *S. officinalis* from different locations of Spanish and Portuguese waters were used as a reference. Also, 29 individuals from 20 other cephalopod species of 11 genera from the Instituto de Investigaciones Marinas (IIM-CSIC) own tissue collection were included for the specificity assay (Table 1). All reference individuals had a known origin and were identified visually prior to the FINS identification. Additionally, 16 commercial samples were collected from supermarkets and restaurants in Galicia region (Spain) for the application to commercial products (Table 2). All tissue samples were stored at −20 °C until analysis.

A portion of 0.3 g of muscle tissue from each sample was digested at 56 °C in a thermo shaker with 860 µL of lysis buffer (1% Sodium Dodecyl Sulfate (SDS), 150 mM NaCl, 2 mM Ethylenediaminetetraacetic acid (EDTA) and 10 mM Tris-HCl at pH 8), 100 µL of guanidinium thiocyanate 5 M and 40 µL of proteinase K (20 mg/mL). After 3 h, 40 µL of extra proteinase K was added and left overnight. DNA was isolated with the Wizard DNA Clean-up System kit (Promega, Madison, WI, USA) following the manufacturer’s protocol. Double-stranded DNA obtained was quantified with Qubit dsDNA BR Assay Kit (Life Technologies, Carlsbad, CA, USA) and Qubit 3.0 fluorometer (Invitrogen, Carlsbad, CA, USA). Purified DNA was stored at −20 °C until further analysis.

### 2.2. FINS Identification of Samples

Reference and commercial samples were authenticated by FINS (forensically informative nucleotide sequencing) in order to test the reliability of the method developed. PCR reactions were carried out in a Verity 96 wells Thermal cycler (Applied Biosystems, Foster City, CA, USA) with Illustra PuReTaq Ready-To-Go PCR Beads (GE Healthcare, Chicago, IL, USA), 1 µL of each primer (10 µM) and 100 ng of template DNA in a final volume of 25 µL. Primers designed by Folmer [19] LCO1490-5′GGTCAACAAATCATAAAGATATTGG3′ and HCO2198-5′TAAACTTCAGGGTGACCAAAAAATCA3′ were used to amplify a 750 base pairs (bp) fragment of the mitochondrial COI region, with the following thermal protocol: a preheating step of 3 min at 95 °C, followed by 35 cycles of 1 min at 95 °C, 1 min at 40 °C and 1.5 min at 72 °C, with a final extension step at 72 °C for 7 min. When amplification of COI fragment failed, the 16SVAR primers described by Chapela [11] 16SVAR-F- 5′CAAATTACGCTGTTATCCCTATGG3′ and 16SVAR-R- 5′GACGAGAAGACCCTAATGAGCTTT3′ were used to amplify a 210 bp fragment of the mitochondrial 16S rDNA, with the thermal protocol as follows: a preheating step of 3 min at 95 °C, followed by 35 cycles of 40 s at 94 °C, 40 s at 50 °C and 40 s at 72 °C, with a final extension step at 72 °C for 7 min. Negative and positive controls were included in all PCR sets.

Primers designed in this study for the minor groove binding (MGB)-TaqMan assay were also used for FINS identification in 3 cases of processed commercial samples of *Sepia* spp. (cooked and canned), where both COI and 16S sets of primers failed to amplify, with the following thermal protocol: a preheating step of 3 min at 95 °C, followed by 35 cycles of 40 s at 95 °C, 40 s at 40 °C and 40 s at 72 °C with a final extension step at 72 °C for 7 min. PCR amplicons were visualized on a 2% agarose gel, using UV transillumination (BioRad, Hercules, CA, USA).

PCR products were purified with Illustra ExoProStar (GE Healthcare, Chicago, IL, USA) and sequencing reactions were performed with BigDye Terminator 1.1 (Applied Biosystems, Foster City, CA, USA), following the manufacturer’s instructions. The automatic sequencing was carried out in an ABI PRISM 3130 (Applied Biosystems, Foster City, CA, USA). After automatic sequencing, F and R files were edited with Chromas and aligned with Bioedit [20] to obtain the complete sequence of the fragment. Bioedit software was also used to align the resulting sequence with reference ones from the NCBI and the IIM-CSIC sequence database, which consists of more than 2000 sequences from fish and mollusks specimens that have been collected during 30 years; most of these specimens were morphologically identified and also genetically authenticated. This alignment was imported with MEGA [21] for phylogenetic analysis. The phylogenetic model used for constructing the neighbor-joining tree was Tamura–Nei, with 1000 bootstrap replicates. The results were also authenticated with BLAST [22]. The multiple alignments and the BLAST tool were also used to check the quality and coverage of the resulting sequences.

The COI sequences obtained for reference and commercial samples of this study were uploaded to Genbank [23] (accession numbers: MN977128 to MN977135, MN977138, MN977143, MN977144, MN977146, MN977147, MN977149, MN977152, MN977154 to MN977156, MN977158, MN977159, MN977161 to MN977171, MN977173 to MN977177, MN977179 to MN977191).

### 2.3. RT-PCR Design

**Figure 1:**
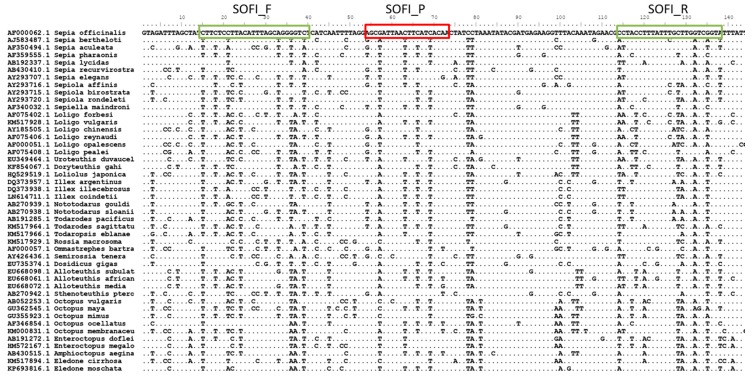
Multiple sequence alignment of the mitochondrial COI (Cytochrome Oxidase I) fragment, showing the position of the primers and probe designed.

In order to find a suitable fragment to design a short and specific system, a large number of nuclear and mitochondrial cephalopod sequences from public and IIM-CSIC databases were aligned and analyzed. A fragment of the COI region was suitable for the design of an MGB-Taq-Man Primers and Probe set, complying with the requirements of showing low intraspecific variability and high interspecific variability and allowing the amplification of a short fragment (122 bp, primers included). The sequences of primers (F and R) and Probe (P) are the following (see Figure 1):

SOFI_F: 5′CTTCTCCTTACATTTAGCWGGRGTCT3′

SOFI_R: FAM-5′TACCGAYCAAGCAAATAAAGGTAGG3′-MGB

SOFI_P: 5′AGCGATTAACTTCATCA3′

### 2.4. Real-Time PCR Conditions and Data Treatment

Concentrations of 50, 300 and 900 nM of each primer and 25, 50, 75, 100, 125, 150, 175, 200 and 225 nM of the probe were tested in order to select the optimal reaction conditions. The combination that gave the lowest threshold cycle (Ct) value and the highest final fluorescence was selected for the subsequent assays. The selected concentrations were 300 nM of SOFI_F primer, 900 nM of SOFI_R primer and 150 nM of SOFI_P probe.

Thus, each 20 µL reaction contained 10 µL of TaqMan Fast Universal Master Mix (2X), No AmpErase UNG (Applied Biosystems, Foster City, CA, USA), 1 µL of Primer SOFI_F (6 µM), 1 µL of Primer SOFI_R (18 µM), 1 µL of Probe SOFI_P (3 µM) and 100 ng of template DNA. Reactions were amplified in a 7500 fast real-time PCR System (Applied Biosystems, Foster City, CA, USA), with the fast ramp speed protocol: 95 °C for 20 s, followed by 40 cycles of 95 °C for 3 s and 60 °C for 30 s. Samples were analyzed in triplicate, and Ct mean and standard deviation of each individual were registered.

## 3. Results

### 3.1. Efficiency and Detection Limit

Different quantities of template DNA of *S. officinalis*, from 10^−5^ ng to 100 ng were used for the efficiency assay. Over this range of dilutions, the response was linear with a slope of −3.13, an *R*^2^ of 0.999 and an efficiency of 101%, following the equation: E = 10^−1/b^ − 1 [24]. The acceptable efficiency values range from 90% to 110%, therefore, 101% can be considered ideally optimal. The minimum quantity of DNA detected was 10^−4^ ng. The automatic threshold generated in this assay was 0.02, the value used in the subsequent analyses.

### 3.2. Inclusivity and Specificity

**Figure 2:**
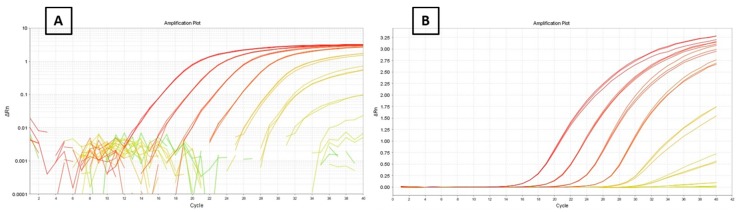
Amplification plots of the 10X dilution series of *Sepia officinalis* DNA (**A**): logarithmic, (**B**): linear.

A total of 14 samples of *S. officinalis* from different locations and dates of capture were tested (Table 1), obtaining Ct data between 12.59 and 16.16, with a Ct mean of 14.04 (Figure 2A).

**Figure 3:**
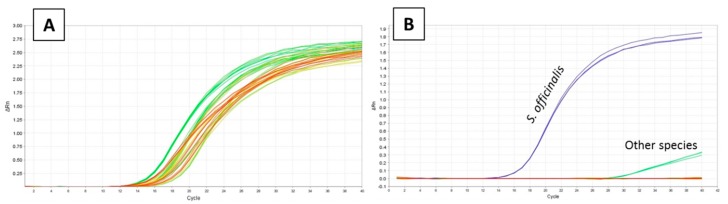
(**A**) Inclusivity test: amplification pattern of reference samples of *Sepia officinalis*. (**B**) Specificity test: amplification pattern of reference samples of *Sepia officinalis* and the rest of the species tested.

In the other 19 species tested (Table 1), none of them presented any fluorescence signal with the exception of one specimen of *Loligo vulgaris*, which showed a late amplification signal (Figure 3B). In view of these results, another specificity assay was carried out with seven additional individuals of *L. vulgaris*, obtaining a Ct mean of 34.0, a result that is significantly different from the Ct of *S. officinalis* when a mean comparison test (one way ANOVA) was run (*p* < 0.001).

### 3.3. Application to Commercial Products

According to the Spanish regulations for the labeling of fresh, frozen and refrigerated fishery products, the commercial name “Sepia”, “Choco” or “Jibia” is only accepted for products containing *S. officinalis*, while the commercial name “Sepias” can be used for all species of the genus *Sepia* [25]. In the same way, the commercial name “Jibia” or “Sepia” can be only applied to canned products containing the species *S. officinalis* [26]. Therefore, the system was also tested with 16 commercial samples labeled as “Sepia”, “Choco” or “Sepias”, from supermarkets and restaurants of Galicia (Spain), with different degrees of processing such as thawed, frozen, grilled, cooked and canned.

Following the above-mentioned criteria, the FINS identification results of this study revealed four cases of mislabeling regarding species (Table 2), all being substitutions between different species of the genus *Sepia*, constituting a mislabeling rate of 25%. The substitute species found were *Sepia pharaonis*, *Sepia aculeata*, *Sepia bertheloti* and a non-identified species. In four cases, it was not possible to reach the species level with the FINS identification, due to the lack of reference sequences in public databases, but authors could determine that these samples did not belong to *S. officinalis* species by analyzing the results of the neighbor-joining tree and the BLAST tool. The MGB TaqMan real-time PCR system worked in fresh and processed samples of *S. officinalis*, and the method was able to differentiate between products containing *S. officinalis* (Ct mean 15.23) and products containing other species of the Sepiidae family (Ct mean 33.82), with statistical significance (*p* < 0.001). The type of processing did not affect the Ct values, and a good differentiation was obtained both in fresh and frozen products as well as in highly processed samples, such as canned.

The Ct results obtained for both reference and commercial samples containing *S. officinalis* ranged from 12.59 to 17.88, with a Ct mean of 14.40, while the rest of species remained undetected or showed late amplification, with Ct values of 23.62 and higher and a mean Ct of 33.40 and this Ct mean resulted significantly different from the Ct of samples containing *S. officinalis* (*p* < 0.001).

## 4. Discussion

Results confirm TaqMan real-time PCR technique as a powerful tool for species authentication, due to its characteristics of specificity, increased with Minor Groove Binding technology (MGB probes) [27], and its sensitivity, allowing the detection of very low quantities of target DNA. Real-time PCR also allows the detection and quantification of target DNA in one step, eliminating post-PCR steps and saving labor time. The method described in this work includes these characteristics of specificity, sensitivity and fastness, since the real-time PCR analysis takes around 40 min, which means that, depending on the tissue digestion protocol, the complete analysis from the tissue sample can be carried out in 3–4 h. This feature and the reduced equipment needed, opens the possibility of the optimization of the method for analyses on-site at the different levels of the value chain, including the point of sale. The cost of the analysis (less than 5 euros per sample) is also much lower than sequencing-based methods, which makes it affordable for low-resources control units.

*S. officinalis* is marketed under several types of processing, including those that eliminate the characteristics for visual identification, such as peeling, cutting, cooking and canning. This makes these products vulnerable to species substitution, intentional or not. Results also show that the design of the primer set allows the amplification and authentication of the species even in samples where processing may lead to DNA degradation and/or fragmentation, such as canning. The lack of rapid methods for this task makes the control of this market laborious and costly, and this technique emerges as the only available alternative at the moment.

The sampling at supermarkets has also revealed that Spanish legislation of the commercial names in canned products needs to be updated since it has not been reviewed since 1986. Taking into account the current legislation, canned products are not obliged to show the scientific name on the labels, i.e., commercial names such as “Sepia” can be found, which correspond to several species. The authors consider that this system is no longer suitable for the current market, where the amount of cephalopod species in the market has greatly increased while different species may achieve significant differences in market price.

The Ct values obtained in this study for the target species are at the same level or lower than other recent works using the TaqMan real-time PCR technique for species identification [28,29]. The significant differences found between the data corresponding to *S. officinalis* and the other species prove that Ct values can be used to determine whether a sample contains *S. officinalis* or another cephalopod species. Results also prove the high specificity of the system, which works for the differentiation of *S. officinalis* from the other species of the genus *Sepia* with commercial importance, demonstrating the utility of the method in food control, since the reported cases of mislabeling in the family Sepiidae show substitutions between species belonging to the same genus, as shown in previous publications [9,10] and confirmed in this study. Although the system has not been tested with all the species of the genus *Sepia*, this study included those with relevance to the market. Nonetheless, further analysis could be carried out to confirm the specificity of the method with other species of the genus *Sepia* which might have some commercial relevance in certain countries. The level of mislabeling found in this work (25%) is slightly lower than those found in the aforementioned articles, but still in the range of significant mislabeling. However, the different sampling procedures do not allow an adequate comparison, therefore, authors cannot affirm that there has been a decrease in the mislabeling rates. Nevertheless, these results highlight the need for an effective tool for the control of this type of product.

## 5. Conclusions

As a conclusion, this work presents a rapid, non-expensive and reliable method, able to differentiate *S. officinalis* from other species of the genus *Sepia* and other cephalopod species in food samples with different levels of processing, making it useful for food control authorities in the whole food value chain.

This study also found a moderate level of mislabeling in Sepia products, which highlights the need for more efficient control of the authenticity of this type of product.

## Figures and Tables

**Table 1:** Reference samples used in this study and threshold cycle (Ct) results.

Sample Code	Species	Common Name (FAO)	Geographic Origin	Ct Mean ± SD
SOFF2	*Sepia officinalis*	Common cuttlefish	Atlantic, Northeast (FAO 27.9) Vigo	12.98 ± 0.32
SOFF3	*Sepia officinalis*	Common cuttlefish	Atlantic, Northeast (FAO 27.9) Cambados	14.05 ± 0.26
SOFF4	*Sepia officinalis*	Common cuttlefish	Atlantic, Northeast (FAO 27.9) Cambados	14.80 ± 0.04
SOFF5	*Sepia officinalis*	Common cuttlefish	Atlantic, Northeast (FAO 27.9) Cambados	16.16 ± 0.35
SOFF6	*Sepia officinalis*	Common cuttlefish	Atlantic, Northeast (FAO 27.9) Vigo	13.57 ± 0.70
SOFF7	*Sepia officinalis*	Common cuttlefish	Atlantic, Northeast (FAO 27.9) Vigo	15.37 ± 0.10
SOFF8	*Sepia officinalis*	Common cuttlefish	Atlantic, Northeast (FAO 27.9) Vigo	13.51 ± 0.07
SOFF9	*Sepia officinalis*	Common cuttlefish	Atlantic, Northeast (FAO 27)	14.11 ± 0.20
SOFF10	*Sepia officinalis*	Common cuttlefish	Atlantic, Northeast (FAO 27)	15.65 ± 0.21
SOFF11	*Sepia officinalis*	Common cuttlefish	Atlantic, Northeast (FAO 27)	13.86 ± 0.48
SOFF12	*Sepia officinalis*	Common cuttlefish	Atlantic, Northeast (FAO 27)	13.25 ± 0.39
SOFF15	*Sepia officinalis*	Common cuttlefish	Atlantic, Northeast (FAO 27.9) Algarve	12.72 ± 0.06
SOFF16	*Sepia officinalis*	Common cuttlefish	Atlantic, Northeast (FAO 27.9) Algarve	12.59 ± 0.19
SOFF17	*Sepia officinalis*	Common cuttlefish	Atlantic, Northeast (FAO 27.9) Algarve	13.98 ± 0.12
SBER 2	*Sepia betheloti*	African cuttlefish	Atlantic, Eastern Central (FAO 34)	≥40
SBER 3	*Sepia betheloti*	African cuttlefish	Atlantic, Eastern Central (FAO 34)	≥40
SORB 4	*Sepia orbygniana*	Pink cuttlefish	Atlantic, Northeast (FAO 27)	≥40
SORB 5	*Sepia orbygniana*	Pink cuttlefish	Atlantic, Northeast (FAO 27)	≥40
SPHA 1	*Sepia pharaonis*	Pharaon cuttlefish	Indian Ocean, Western (FAO 51)	≥40
LVUL 2	*Loligo vulgaris*	European squid	Western Central Atlantic (FAO 31)	27.00 ± 0.16
LVUL 1	*Loligo vulgaris*	European squid	Atlantic, Northeast (FAO 27)	26.03 ± 0.28
LVUL 5	*Loligo vulgaris*	European squid	Atlantic, Northeast (FAO 27)	29.15 ± 0.14
LVUL 3	*Loligo vulgaris*	European squid	Western Central Atlantic (FAO 31)	≥40
LVUL 4	*Loligo vulgaris*	European squid	Western Central Atlantic (FAO 31)	≥40
LVUL 6	*Loligo vulgaris*	European squid	Western Central Atlantic (FAO 31)	≥40
LVUL 7	*Loligo vulgaris*	European squid	Western Central Atlantic (FAO 31)	≥40
LVUL 8	*Loligo vulgaris*	European squid	Western Central Atlantic (FAO 31)	29.80 ± 0.40
LREY 1	*Loligo reynaudi*	Cape Hope squid	Atlantic, Southeast (FAO 47)	≥40
IILL 2	*Illex illecebrosus*	Northern Shortfin squid	Atlantic, Northwest (FAO 21)	≥40
TEBL 1	*Todaropsis eblanae*	Lesser flying squid	Atlantic, Northeast (FAO 27)	≥40
TPAC 3	*Todarodes pacificus*	Japanese flying squid	Pacific, Northwest (FAO 61)	≥40
ICOI 10	*Illex coindetii*	Southern shortfin squid	Atlantic, Northeast (FAO 27)	≥40
LGAH 9	*Loligo gahi*	Patagonian squid	Pacific, Southeast (FAO 87)	≥40
MHYA 8	*Martialia hyadesi*	Sevenstar flying squid	Atlantic, Antarctic (FAO 48)	≥40
NSLO6	*Nototodarus sloanii*	Wellington flying squid	Pacific, Southwest (FAO 81)	≥40
TSAG 1	*Todarodes sagittatus*	European flying squid	Atlantic, Northeast (FAO 27)	≥40
OVUL 142	*Octopus vulgaris*	Common octopus	Atlantic, Northeast (FAO 27)	≥40
OCYA 3	*Octopus cyanea*	Big blue octopus	Pacific, Western Central (FAO 71)	≥40
OCYA 4	*Octopus cyanea*	Big blue octopus	Pacific, Western Central (FAO 71)	≥40
OMIM 1	*Octopus mimus*	Changos octopus	Pacific, Southeast (FAO 87)	≥40
ECIR 143	*Eledone cirrhosa*	Horned octopus	Atlantic, Northeast (FAO 27)	≥40
DGIG 1	*Dosidicus gigas*	Jumbo squid	Pacific, Southeast (FAO 87)	≥40
AMEM 1	*Amphioctopus membranaceus*	Webfoot octopus	Indian Ocean, Western (FAO 51)	≥40

FAO: Food and Agriculture Organization. SD: Standard Deviation.

**Table 2:** Commercial samples used for validation. The mislabeled samples are highlighted in red.

Sample Code	Type of Processing	Type of Establishment	Species Declared	Species Identified by FINS	Ct Mean ± SD
S1	Frozen	Supermarket	*Sepia spp.*	*Sepia pharaonis*	29.77 ± 0.62
S2	Frozen	Supermarket	*Sepia spp.*	*Sepia pharaonis*	27.68 ± 0.06
S3	Frozen	Supermarket	*Sepia spp.*	*Sepia sp* (not *S. officinalis*)	≥40
S4	Frozen	Supermarket	“Sepia”	*Sepia sp* (not *S. officinalis*)	31.85 ± 0.26
S5	Canned	Supermarket	“Sepia”	*Sepia officinalis*	16.91 ± 0.47
S6	Frozen	Supermarket	*Sepia spp.*	*Sepia sp* (not *S. officinalis*)	≥40
S7	Cooked	Supermarket	*Sepia officinalis*	*Sepia officinalis*	17.88 ± 0.94
S8	Canned	Supermarket	“Sepia”	*Sepia officinalis*	15.41 ± 0.03
S10	Grilled	Restaurant	“Choco”	*Sepia officinalis*	13.70 ± 0.06
S11	Frozen	Supermarket	*Sepia aculeata*	*Sepia sp* (not *S. officinalis*)	≥40
S12	Frozen	Supermarket	*Sepiella spp.*	*Sepiella inermis*	≥40
S13	Frozen	Supermarket	*Sepia pharaonis*	*Sepia aculeata*	≥40
S14	Thawed	Supermarket	*Sepia officinalis*	*Sepia officinalis*	14.03 ± 0.28
S15	Grilled	Restaurant	“Sepia”	*Sepia bertheloti*	26.08 ± 0.11
S16	Thawed	Supermarket	*Sepia officinalis*	*Sepia officinalis*	13.43 ± 0.06
S17	Canned	Supermarket	“Sepia”	*Sepia pharaonis*	23.62 ± 0.23

FINS: Forensically Informative Nucleotide Sequencing.
